# COVID-19 and brain-heart-lung microbial fingerprints in Italian cadavers

**DOI:** 10.3389/fmolb.2023.1196328

**Published:** 2023-06-14

**Authors:** Gulnaz T. Javan, Sheree J. Finley, Matteo Moretti, Silvia D. Visonà, Melissa P. Mezzari, Robert L. Green

**Affiliations:** ^1^ Department of Physical and Forensic Sciences, Alabama State University, Montgomery, AL, United States; ^2^ Department of Public Health, Experimental and Forensic Medicine, University of Pavia, Pavia, Italy; ^3^ Alkek Center for Metagenomics and Microbiome Research, Baylor College of Medicine, Houston, TX, United States

**Keywords:** COVID-19, *postmortem microbiome*, internal organs, 16S rRNA, cadavers, thanatomicrobiome

## Abstract

**Introduction:** The fact that SARS-CoV-2, the coronavirus that caused COVID-19, can translocate within days of infection to the brain and heart and that the virus can survive for months is well established. However, studies have not investigated the crosstalk between the brain, heart, and lungs regarding microbiota that simultaneously co-inhabit these organs during COVID-19 illness and subsequent death. Given the significant overlap of cause of death from or with SARS-CoV-2, we investigated the possibility of a microbial fingerprint regarding COVID-19 death.

**Methods:** In the current study, the 16S rRNA V4 region was amplified and sequenced from 20 COVID-19-positive and 20 non-COVID-19 cases. Nonparametric statistics were used to determine the resulting microbiota profile and its association with cadaver characteristics. When comparing non-COVID-19 infected tissues versus those infected by COVID-19, there is statistical differences (*p* < 0.05) between organs from the infected group only.

**Results:** When comparing the three organs, microbial richness was significantly higher in non-COVID-19-infected tissues than infected. Unifrac distance metrics showed more variance between control and COVID-19 groups in weighted analysis than unweighted; both were statistically different. Unweighted Bray-Curtis principal coordinate analyses revealed a near distinct two-community structure: one for the control and the other for the infected group. Both unweighted and weighted Bray-Curtis showed statistical differences. Deblur analyses demonstrated Firmicutes in all organs from both groups.

**Discussion:** Data obtained from these studies facilitated the defining of microbiome signatures in COVID-19 decedents that could be identified as taxonomic biomarkers effective for predicting the occurrence, the co-infections involved in its dysbiosis, and the evolution of the virus.

## Introduction

In the era of COVID-19, accurate autopsies are crucial to determine the cause of death in decedents who test positive for SARS-CoV-2. Given the substantial pathological commonalities of cause of death *from* or *with* SARS-CoV-2, we investigated the possibility of a microbial fingerprint in organs from SARS-CoV-2 deaths. A human corpse subsists as a specialized disturbance habitat that selects for a distinct thanatomicrobiome structure capable of decomposing the host depending on the cause of death and abiotic and biotic factors surrounding the death (Can et al., 2014; Javan et al., 2016; Kaszubinski et al., 2020). SARS-CoV-2 binds to angiotensin-converting enzyme 2 (ACE2) receptors present on the surface of various cells in the body and can negatively affect essentially all organs of the host. Before death, there is strong evidence that COVID-19 affects the brain structure (Douaud et al., 2021), heart (Delorey et al., 2021), lungs (Elezkurtaj et al., 2021), and gut microbiome (Yeoh et al., 2021). Many *postmortem* molecular questions regarding the pathophysiology of COVID-19 infection have not been elucidated yet.

Brain imaging has demonstrated degenerative spread of COVID-19 via neuroinflammatory events (Yachou et al., 2020), olfactory pathways involving anosmia (Gori et al., 2020), or loss of sensory input of taste (Ludwig et al., 2022). After death, the brain demonstrates changes due to hypoxia, increased carbon dioxide levels, and cytokine storm (Jain, 2020; Douaud et al., 2021). Furthermore, the principal cause of death in SARS-CoV-2 infection is respiratory failure but cardiac indications also contribute largely to mortality. Studies have shown that abnormal echocardiography were present in up to 55% of all COVID-19 infected cases (Dweck et al., 2020). The contribution of the co-infection of SARS-CoV-2 and other microorganisms in cardiomyocytes remains unclear.

**FIGURE 1:**
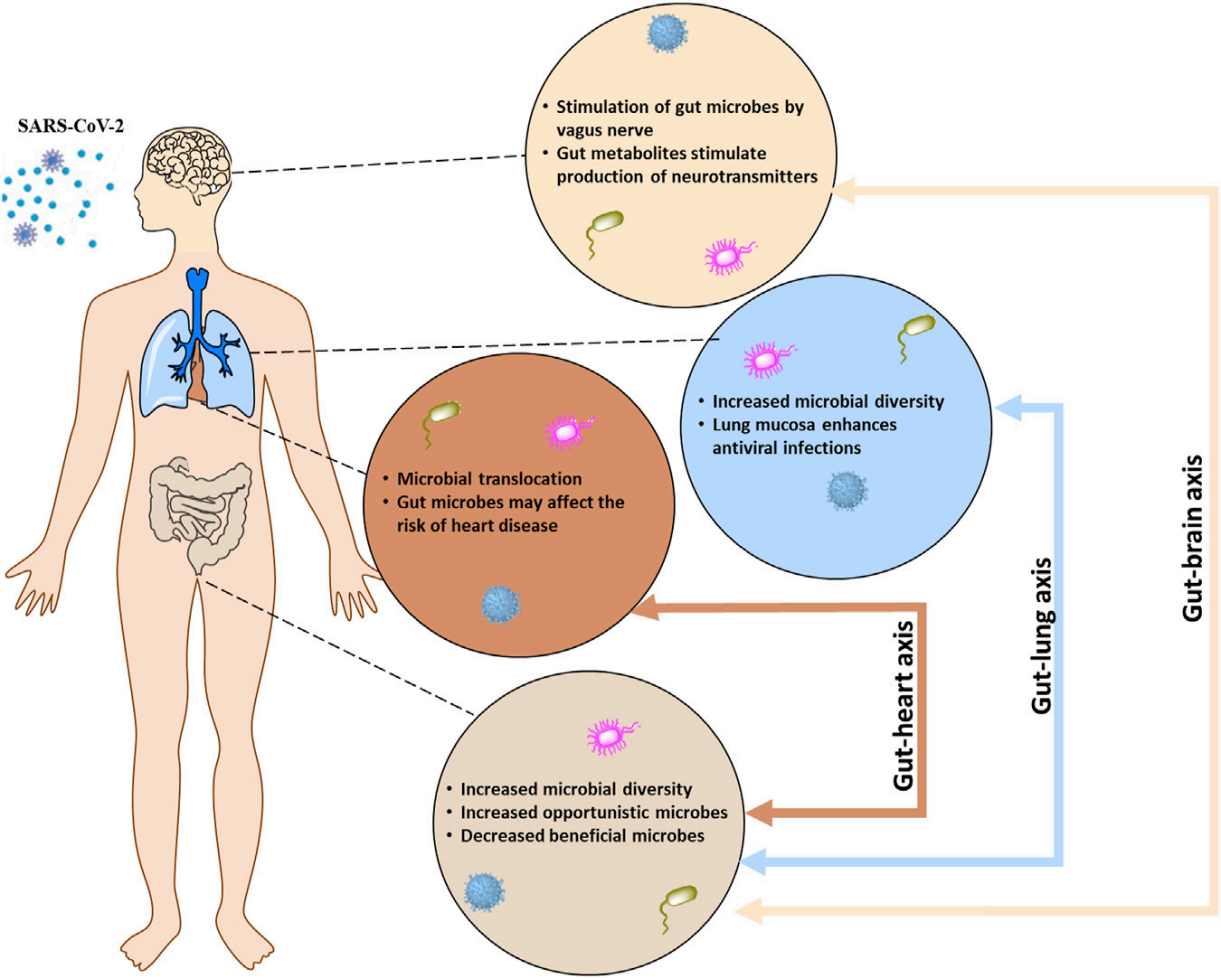
The bidirectional communication between the central nervous system (brain), respiratory system (lung), and cardiovascular system (heart) with the enteric nervous system (gut) occurs through the gut-brain, gut-lung, and the gut-heart system, respectively.

Bacteria and/or bacterial products often have direct and indirectly interactions with viruses that aid in pathogenicity. For example, respiratory syncytial virus (RSV) interacts with *Streptococcus pneumonia*, *Pseudomonas aeruginosa*, and *Haemophilus influenzae* to increase bacterial invasiveness and increase host cell adhesion molecules (Lian et al., 2022). Likewise, intracellular overgrowth of bacteria contributes to intracytoplasmic organelle damage which causes decreased viral antigen production during co-infection (Di Biase et al., 2000). Normal oxidative and inflammatory molecular pathways in the brain, heart, and lungs can drastically change during COVID-19 illness which could potentially allow proliferation of microorganisms and subsequent damage to organs. The current study seeks to ascertain the possibility of a microbial fingerprint in the brain, heart, and lungs related to SARS-CoV-2 death (Figure 1).

Microorganisms deliver specific fingerprints that differ from person to person. Regarding distinguishing microbes, often it is crucial to differentiate them between their taxonomic classes, which is called microbial fingerprinting. This study demonstrates the potentiality of a microbial fingerprint and its use in crime scenes parallel to conventional fingerprinting. For example, can *postmortem* microbial fingerprint characterization be used in medicolegal investigations to link physical evidence to the criminal or victim (Fierer et al., 2010; Aaspõllu et al., 2011; Nishi et al., 2015)?

The distinct thanatomicrobiome profiles in organs from human cadavers that died of or with SARS-CoV-2 may help guide the application of forensic microbiology tools to establish the real cause of death. The H_0_ (null hypothesis) of our study is that there are no differences in the microbiome of COVID-19 and non-COVID-19 cadavers. Thus, the H_A_ (alternative hypothesis) is that there are differences in the microbiome of COVID-19 and non-COVID-19 cadavers. To test the alternative hypothesis, we used a two-pronged strategy to first, establish an *in vitro* model of detection of microbial diversity in brain, heart, and lung tissue microbiota from 40 human cadavers, half of them infected with SARS-CoV-2.

The chi-squared test was used to determine if the deviations in the data are caused by sampling or experimental error. Deviations may be caused by random chance or if there are differences in our data that are due to a statistically significant difference. If they were caused by a statistically significant difference, it gives evidence to support or reject our hypothesis. Generally, when approaching a chi square test, you start with the null hypothesis that there is no difference in the data; that is, that any differences are in fact due to sampling errors, experimental errors, or by chance errors. So, by calculating chi-squared values generate statistical support for whether to reject or accept the hypothesis. The second approach involved adjusting all *p*-values for multiple comparisons with the FDR algorithm to control the number of false discoveries in those tests that result in a discovery (i.e., a significant result). It has greater ability (i.e., power) to find truly significant results.

The results demonstrated that microbial profiles vary significantly during COVID-19 infection as the corpse decomposes. When comparing non-COVID-19 infected samples versus COVID-19 infected samples, there is statistical difference between the brain, heart, and lung from the infected group only. Among the three organs, microbial richness was significantly higher in the non-COVID-19-infected tissues compared to the infected tissues. Further, Unifrac distance analyses demonstrated that there was more variance between control and COVID-19 groups in weighted analysis than unweighted; both were statistically different. Also, unweighted Bray-Curtis principal coordinate analyses showed a near distinct two-community structure: one for control and the other for the COVID-19-infected group. Both unweighted and weighted Bray-Curtis showed statistical differences. These results facilitated the defining of microbiome profiles in COVID-19 decedents that could be identified as taxonomic biomarkers effective for predicting the occurrence, dysbiosis, and evolution of the virus.

## Results

### Impact of COVID-19 and microbial diversity

To estimate β-diversity, un-weighted and weighted UniFrac distances, as well as Bray-Curtis dissimilarity, were calculated from the amplicon sequence variants (ASVs) and genera relative abundance tables were generated*.*

**FIGURE 2:**
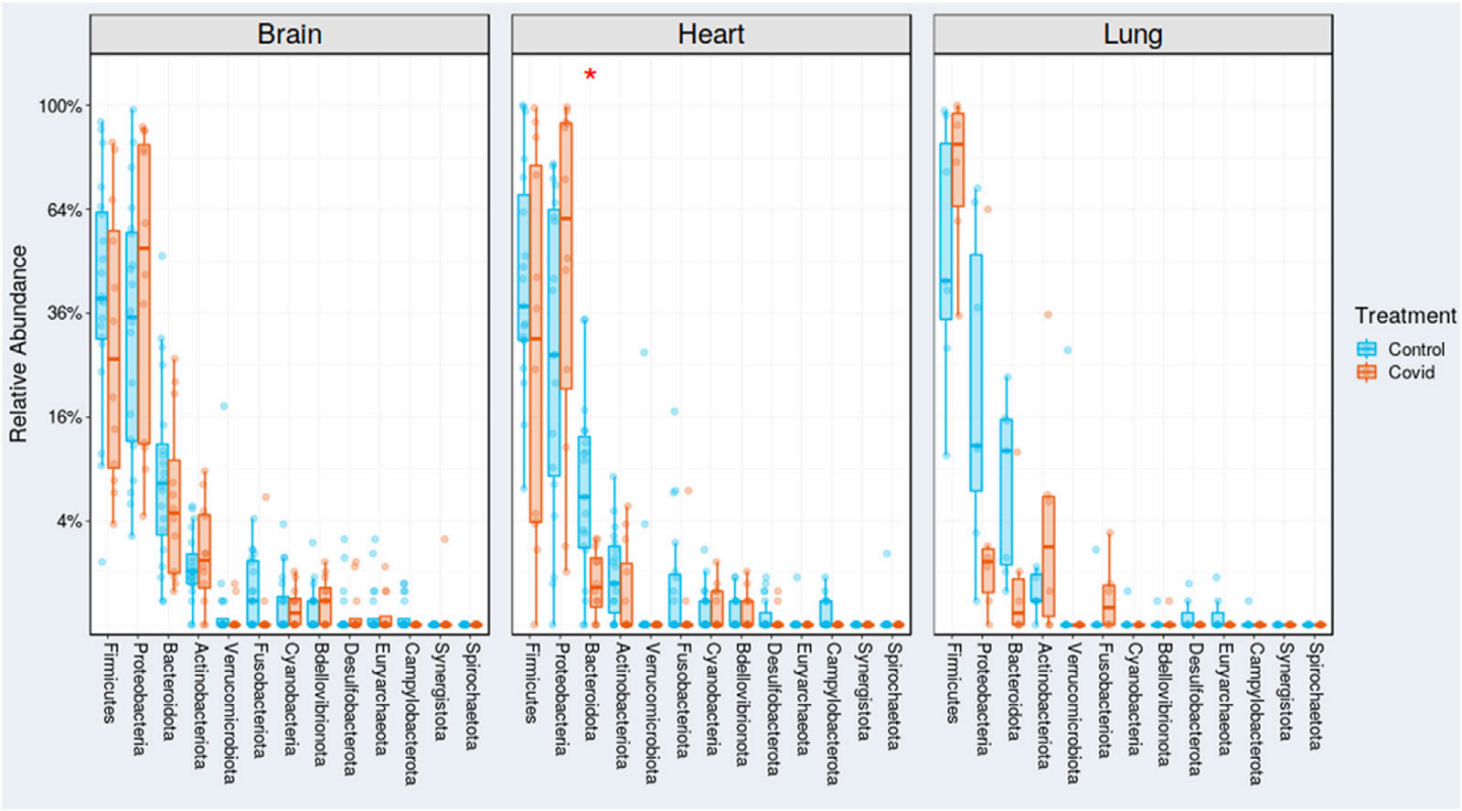
Relative abundances of unweighted and weighted UniFrac distances of COVID-19 (red) and non-COVID-19 (blue) cases. Red and blue boxes delineate the interquartile range (IQR), and the whiskers extend to 1.5  ×  IQR. Outliers are depicted as points.

The Firmicute, *S. aureus*, demonstrated enrichment in all cases, specifically COVID-19-infected lungs (Figure 2). Likewise, the Actinobacteria, *Corynebacterium* was the next highest in abundance in the COVID-19-infected lungs.

**FIGURE 3:**
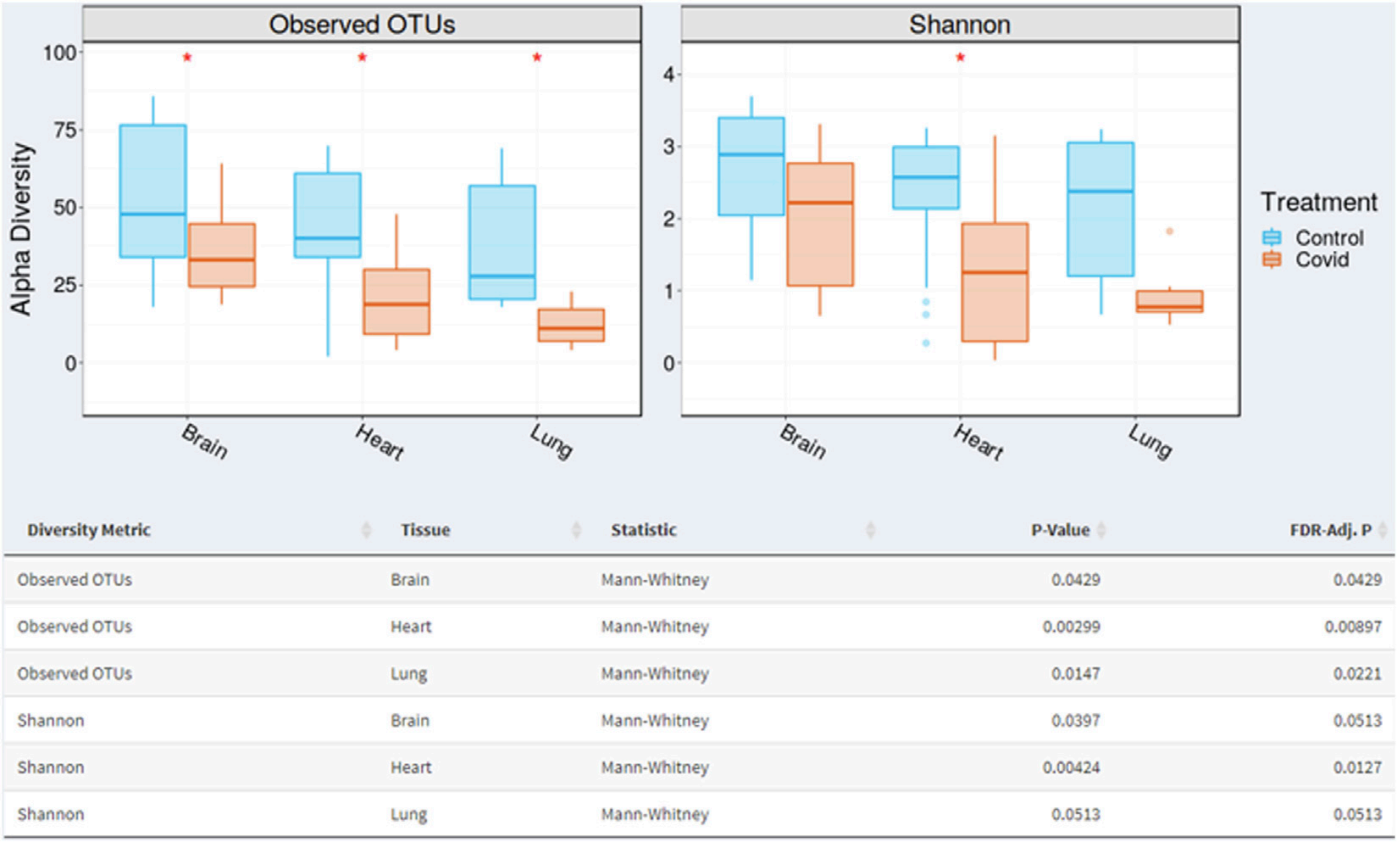
Alpha diversity varied significantly (*p* < 0.05; Mann-Whitney test) between organs (brain, heart, and lung). The box plots demonstrate where the 25% and 75% quartile boundaries are, while the central, thick line is the 50% quartile (median). The “whiskers” on the outside of the plot show where the smallest and largest values are. Consequently, each quarter of the box plots contain approximately 25% of samples.

The results also revealed that there are statistical differences between organs from the COVID-19 infected group. At the operational taxonomic unit (OTU) level, alpha diversity presented significant differences between organs (adjusted *p*-value < 0.05, Mann-Whitney test). Controls showed higher OTU richness values in all organs than COVID-19 (statistically significant). Shannon index of microbial richness and evenness values in all organs than COVID-19 (only heart showed statistical differences at *p*-value < 0.005). Based on the Shannon diversity index, there is no statistical differences when comparing COVID-19 and control. Shannon index of microbial richness and evenness values in all organs than COVID-19 (only heart showed statistical differences) (Figure 3).

**FIGURE 4:**
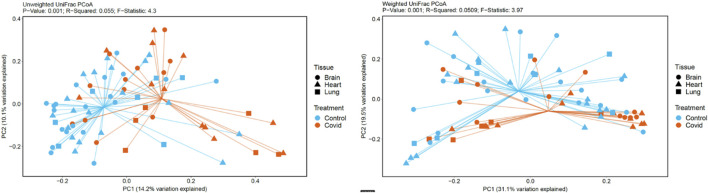
PCoA plots generated based on unweighted and weighted Unifrac distances metrics.

For unweighted UniFrac, there was relatively low variance between control and COVID-19, with only 14.2% of variance explained by PC1 Axis and 7.27% explained by PC2 Axis (Figure 4). For weighted UniFrac, there was more variance between control and COVID-19 compared to unweighted UniFrac PCoA, with 31.17% of the variance explained by PC1 Axis and 19.5% explained by PC2 Axis.

*Staphylococcus aureus* was the most abundant bacteria found in COVID-19-infected lung tissue (Supplementary Figure S1). The highest percentage of bacteria in heart tissue on the genera level was Enterobacteriaceae, which occurred in both groups (Supplementary Figure S2). From the 12 samples of the COVID-19 group, three showed increased relative abundance of specific genera, *Enterococcus*, *Enterobacter*, and *Lactobacillus*. From the 19 samples of the control group, four showed increased relative abundance of *Hathewaya*, *Clostridium*, *Paeniclostridium*, and *Morganella*. *Escherichia* and *Shigella* were the most abundant bacteria found in COVID-19-infected heart tissue (Supplementary Figure S3). These two bacteria are closely related and the 16S rRNA gene comparison does differentiate between *E. coli* and *Shigella* spp. as a result of greater than 99% sequence identity (Ragupathi et al., 2018).

### Deblur analyses

There is statistical difference in taxonomic distribution at the phylum level between organs from COVID-19 and non-COVID 19 groups. The *p*-values for Deblur analyses were calculated from the chi-squared test. A total of 28 Firmicutes genera (which included two *Clostridium* species) were detected on brain, heart, and lung tissues. Firmicutes is listed in all comparisons at the phylum level (Table 1). The lungs show statistical differences for three bacterial phyla: *Bacterioidota*, *Firmicutes*, and *Proteobacteria*. The heart shows statistical differences for seven bacterial phyla: *Actinobacteriota, Campylobacterota, Cyanobacteria, Firmicutes, Fusobacteriota, Myxococcota,* and *Verrucomicrobiota*. The brain shows statistical differences for six bacterial phyla: *Bacterioidota, Campylobacterota,* Deinococcota, *Firmicutes, Fusobacteriota,* and *Proteobacteria*.

**TABLE 1:** The *p*-values from chi-squared test showing statistical differences at the phylum level.

Phylum	Brain	Heart	Lung
Bacteria; *Actinobacteriota*		<0.05	
Bacteria; *Bacteroidota*	<0.05		<0.05
Bacteria; *Campylobacterota*	0.022	0.008	
Bacteria; *Cyanobacteria*		0.029	
Bacteria; *Deinococcota*	<0.05		
Bacteria; *Firmicutes*	<0.05	<0.05	<0.05
Bacteria; *Fusobacteriota*	0.001	0.02	
Bacteria; *Myxococcota*		<0.05	
Bacteria; *Proteobacteria*	<0.05		<0.05
Bacteria; *Verrucomicrobiota*		0.046	

## Discussion

Studies have shown that the gut microbiome composition is significantly altered in patients with COVID-19 compared to non-COVID-19 cases regardless of whether patients had been treated with medication (Yeoh et al., 2021). In the current study, *Staphylococcus aureus* was the most abundant bacteria found in COVID-19-infected lung tissue (Supplementary Figure S1). Studies have demonstrated that the gut microbiome was altered in COVID-19 infections with an enrichment in opportunistic pathogens (Wang et al., 2021). For example, *S. aureus* generally have higher abundance in the lungs of COVID-19-infected patients Thus, *S. aureus* is commonly found in hospital environments for its risk of deadly outcomes such as endocarditis, bacteremia, sepsis, and death. In past viral pandemics, *S. aureus* has been the principal cause of secondary bacterial infections, significantly increasing patient mortality rates (Adalbert et al., 2021). The predominance of *S. aureus* co-infections occurring after patient admission for COVID-19 infection is likely associated with patient interventions identified as intubation and mechanical ventilation, central venous catheter placement, and corticosteroids. In the current study, Gram-positive *S. aureus*, which belongs to the Firmicutes phylum, was shown to be enriched in all cases, especially COVID-19-infected lungs (Figure 2). The gut area has the largest absolute decomposition burden that spreads to the proximate organs, such as the liver and spleen, and extends to the distal organs, such as the heart and brain, depending on the cause of death (Javan et al., 2019). Likewise, the opportunistic bacteria, *Corynebacterium*, was the next highest in abundance in the COVID-19-infected lungs. *Corynebacterium* sp*.* is well known to be a pathogen in lower respiratory tract infection. Studies have reported that ventilator-associated complications (VACs) in COVID-19 patients were due to *Corynebacterium* sp. (Ogawa et al., 2022).

Regarding inflammatory responses that characterize COVID-19 infections, the immune system ceases within 24 h after death. Due to Italian laws, the minimum *postmortem* interval (PMI) for cases in the current study is 24 h. Therefore, due to the extended PMIs, inflammatory components were undetectable in the criminal case cadavers used in this study. Taxa that proliferate in COVID-19 deaths show reduced microbial diversity and an enrichment for microorganisms that can resist inflammatory responses more effectively than others (Hussain et al., 2021). Previous antemortem studies demonstrated that the gut microbiome of non-COVID-19 infected patients had higher abundance of anti-inflammatory bacteria Lachnospiraceae, *Roseburia*, *Eubacterium*, and *Faecalibacterium prausnitzii* compared to the microbiome of patients with COVID-19 (Reinold et al., 2021; Wang et al., 2022). In the current study, as expected these bacteria were not enriched in *postmortem* brain, heart, and lung samples of COVID-19 cases.

The highest percentage of bacteria in heart tissue on the genera level was Enterobacteriaceae, which occurred in both groups (Supplementary Figure S2). From the 12 samples of the COVID-19 group, three showed increased relative abundance of specific genera, *Enterococcus*, *Enterobacter*, and *Lactobacillus*. From the 19 samples of the control group, four showed increased relative abundance of *Hathewaya*, *Clostridium*, *Paeniclostridium*, and *Morganella*. *Escherichia* and *Shigella* were the most abundant bacteria found in COVID-19-infected heart tissue. The highest percentage of bacteria in brain tissue on the genera level was Enterobacteriaceae (*Escherichia* and *Shigella*) in both the control and infected groups (Supplementary Figure S3). *E. coli* are known to translocate from the blood to the central nervous system without apparent damage to the blood–brain barrier, which indicates a transcytosis process (Kaper et al., 2004). *Enterobacter* species are increasingly a cause of nosocomial meningitis among neurosurgery patients. In community-acquired infections, *Enterobacter* was isolated in one of the nine cases of meningitis caused by Gram-negative bacilli (*E. coli* four times, *Klebsiella* species three times, and *Proteus* once) and in five of the 57 episodes of nosocomial meningitis (*E. coli* 17 times, *Klebsiella* species 13 times, *Pseudomonas* species six times, and *Acinetobacter* species six times). *Morganella morganii* is a Gram-negative aerobe, found often as intestinal commensal. It is commonly implicated in urinary tract infections and pyogenic infections, but rarely causes CNS infections especially brain abscess.

Of interest, Deblur analyses showed that Firmicutes (which included two *Clostridium* species) were predominant among all organs from both groups. The detection of *Clostridium* species is accounted for by the *Postmortem Clostridium* Effect (PCE) that distinguishes the rapid proliferation of the species in decaying internal body sites (Javan et al., 2017; Lutz et al., 2020; Javan et al., 2022). These bacteria have adaptive properties that facilitate persistence in anoxic and hypoxic environments that persist until the skin ruptures. Thus, a future research question might include, “Could any of these *postmortem* bacteria, specifically *Clostridium*, be biomarkers across thanatomicrobiome communities derived from different locations in the body?”

## Materials and methods

### Ethics stattement

**FIGURE 5:**
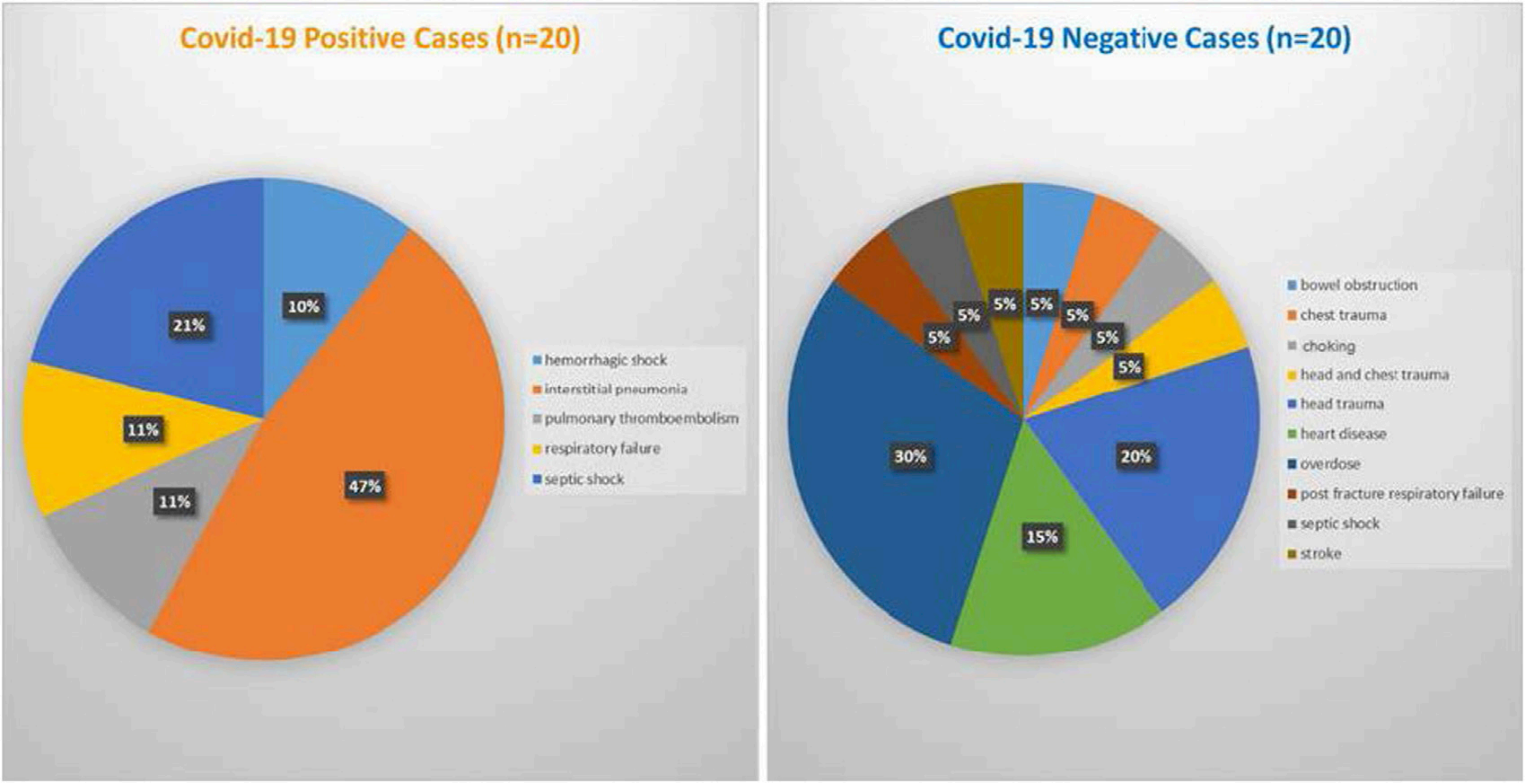
Cause of death for COVID-19 positive cases (left panel) and COVID-19 negative cases (right panel).

*Postmortem* brain, heart, and lung samples were obtained from 40 human cadavers at the Department of Public Health Experimental and Forensic Medicine at the University of Pavia in Pavia, Lombardy, Italy. The study was approved by the Alabama State University Institutional Review Board (IRB) number 2020100. For deceased subjects, consent is not required, because the tissues were collected for forensic purposes, and it is not possible to contact the next of kin under such circumstances. The reference law is authorization n9/2016 of the Guarantor of Privacy, then replaced by REGULATION (EU) 2016/679 OF THE EUROPEAN PARLIAMENT AND OF THE COUNCIL. The causes of death were determined by medical examiner as determined at autopsy with intestinal pneumonia as the most prevalent (47%) among the COVID-19 positive cases and head trauma as the most prevelant (30%) among the control group (Figure 5).

### COVID-19 and cadaver sampling

Corpses were kept in the morgue at 4°C until the time of tissue collection. Tissue sampling was performed in an examination area with an ambient temperature of 20°C. Sections of the internal organs were uniformly dissected using a sterile scalpel and placed in polyethylene bags. Tissue samples were transported from the morgue to Alabama State University on dry ice and immediately frozen at −80°C until futher analyses. Demographic data were collected for each cadaver: age, sex, height, weight, cause of death, and medical history (COVID-19 positive cases Table 2 and non-COVID-19 positive cases; Table 3). The minimum PMI was 24 h and the maximum was 20 days.

**TABLE 2:** Demographic data for COVID-19-negative cases. Age, sex, height, weight, PMI, cause of death, and medical history.

Case	Age	Sex	Height (cm)	Weight (kg)	PMI (day)	Cause of death	Medical history
1	42	M	186	75	4	Overdose	Drug and alcohol abuse
2	48	M	172	85	6	Head trauma	Not reported
3	28	M	172	70	4	Head trauma	Not reported
4	62	M	161	80	1	Heart disease	Arterial hypertension, Obesity
5	63	M	174	70	3	Overdose	HCV+, HIV+, Diabetes, Cardiomyopathy, Drug and alcohol abuse
6	73	F	158	50	7	Heart disease	Not reported
7	41	M	172	92	3	Overdose	Drug and alcohol abuse
8	41	M	180	100	3	Overdose	Drug and alcohol abuse
9	46	F	160	70	2	Bowel obstruction	Psychiatric disorders
10	87	F	159	55	3	Head trauma	Not reported
11	91	F	147	45	11	Septic shock	Chronic obstructive pulmonary disease
12	37	F	170	55	4	Overdose	Drug and alcohol abuse, Psychiatric disorders
13	80	M	162	80	5	Stroke	Diabetes, Arterial hypertension, Chronic liver disease
14	75	F	167	85	8	Overdose	Obesity, Cardiomyopathy, Arterial hypertension
15	74	M	168	70	3	Chest trauma	Lung cancer
16	73	F	165	65	3	Post fracture resp. Failure	Not reported
17	68	M	166	70	4	Head and chest trauma	Aortic aneurysm
18	82	F	161	55	5	Heart disease	Alzheimer disease, Hypertension
19	68	M	163	65	6	Head trauma	Not reported
20	61	M	167	60	5	Choking	Alcohol abuse

**TABLE 3:** Demographic data for COVID-19-negative cases. Age, sex, height, weight, PMI, cause of death, and medical history.

Case	Age	Sex	Height (cm)	Weight (kg)	PMI (day)	Cause of death	Medical history
1	90	F	161	55	11	Interstitial pneumonia	Ictus cerebri, Chronic obstructive pulmonary disease
2	81	F	167	90	15	Interstitial pneumonia	Lewy body dementia
3	83	F	154	45	13	Interstitial pneumonia	Alzheimer, Breast cancer
4	92	M	169	55	6	Interstitial pneumonia	Chronic vascular encephalopathy, Arterial hypertension, Arthrosis, Myocardiosclerosis
5	80	M	178	75	7	Septic shock	Paraplegia in Guillain-Barré Syndrome, Myocardiosclerosis
6	74	F	155	60	7	Interstitial pneumonia	Cognitive impairment
7	99	F	154	55	10	Interstitial pneumonia	Cognitive impairment, Hyperthyroidism
8	82	F	158	55	7	Pulmonary thromboembolism	Cognitive Impairment
9	77	F	167	65	8	Hemorrhagic shock	Arthrosis
10	50	F	165	70	20	Respiratory failure	Not reported
11	73	M	175	80	5	Septic shock	Bladder Cancer - Cystectomy, Chronic renal failure
12	65	M	170	80	7	Interstitial pneumonia	Chronic heart failure
13	72	F	163	50	8	Interstitial pneumonia	Cognitive impairment
14	72	F	158	65	6	Interstitial pneumonia	Not reported
15	84	M	172	55	17	Respiratory failure	COPD, Arterial hypertension, Obstructive sleep apnea syndrome
16	28	M	171	68	7	Hemorrhagic shock	Not reported
17	89	F	145	50	7	Septic shock	Cognitive impairment, Arterial hypertension, Diabetes
18	88	F	130	40	5	Septic shock	Arthrosis, Mental handicap since birth, Recovering alcoholic
19	89	F	150	45	5	Respiratory failure	Cognitive impairment after ictus cerebri, Hiatal hernia
20	90	F	150	40	5	Pulmonary thromboembolism	Arterial hypertension, Atrial fibrillation, Chronic renal failure

### DNA extraction, library preparation, and sequencing

Genomic DNA was extracted from internal organs by physical disruption using the phenol-chloroform method, which is specifically optimized for recovery of microbial DNA from low-yield samples (Can et al., 2014). The quality and quantity of DNA was determined by spectrophotometry (NanoDrop™). The DNA was analyzed by PCR using universal primers (515F/806R).

Brain, heart, and lung microbiota was evaluated by 16S rRNA gene sequencing on an Illumina MiSeq platform using the 2 × 250 bp paired-end at the Alkek Center for Metagenomics and Microbiome Research (CMMR). Primers used for amplification (515F/806R) targeted the V4 region and contained adapters for MiSeq sequencing in conjunction with a single-index molecular barcode on the reverse primer. Resultant read pairs were demultiplexed, formulated by their molecular barcode, and combined using USEARCH v7.0.1090 (Edgar, 2010) and UCHIME (Edgar et al., 2011). UCHIME improves sensitivity and speed of chimera detection. A minimum overlap of 50 bases were allowed with zero mismatches. Merged reads were trimmed at the first base with a Q5 less than five. Reads containing >0.05 expected errors were discarded by a quality filter.

### Microbiome analyses

Instead of operational taxonomic units (OTUs), denoising tools to generate sequence variants were employed. DEBLUR was the denoising tool used to analyze the number of sequencing mismatches, and the quality of the sequences were distinguished between sequencing errors and biological variants (Amir et al., 2017). The tool merged sequences into single sequence variants, also called amplicon sequence variants (ASVs), instead of clusters (Jeske and Gallert, 2022). Deblur subtracts the number of projected error-derived reads from neighboring reads based on their Hamming distance which produces stable ASVs at single-nucleotide resolution. The ASV methods have a substantial advantage over OTU analysis in that OTUs need to be clustered for each data set and thus, are never exactly the same (Callahan et al., 2017). In contrast, ASVs can be compared across data sets. Therefore, for small mismatches, it is implicitly known whether these discrepancies are errors or real sequences. Closely related taxa can be discriminated that otherwise would not be distinguishable with the *de novo* clustering of OTU analysis. ASVs are matched to a reference database similar to OTUs. ASVs that do not correspond to a database are kept as unknown taxa, similar to unknown OTUs. The last step of ASV analysis is to count all the reads matching to the same taxa in the reference database or being assigned to the same ASVs or OTUs.

Relative abundances of taxa were recovered by mapping merged reads using the UPARSE algorithm (Edgar, 2013). Box plots, beta-diversity biplots, principal coordinate analysis (PCoA), hierarchical clustering analyses, and respective statistical analyses were executed in the user interface Agile Toolkit for Incisive Microbial Analyses (ATIMA) developed by the Center for Metagenomics and Microbiome Research at Baylor College of Medicine. ATIMA is a standalone, R-based software suite (R Core Team, 2013) to analyze and visualize the microbiome data sets and identify trends in taxa abundance, alpha-diversity, and beta-diversity with sample metadata.

In order to visualize beta diversity differences in tissues from COVID-19 infected and control cadavers, Bray-Curtis PCoA plots employing Monte Carlo permutation tests were generated based on unweighted (qualitative) and weighted (quantitative) Unifrac distances metrics. These distance metrics took into account relatedness of species to calculate distance and to calculate *p*-values. UniFrac is a distance metric used for comparing microbial communities and is a generic test method that describes whether two or more communities have the same structure. Weighted UniFrac was used to examine quantitative differences in community structure, thereby observing the taxa abundance in the microbiome. Unweighted UniFrac was used to determine qualitative differences in the microbial community, thereby considering only presence or absence of observed taxa. All *p*-values are adjusted for multiple comparisons with the FDR algorithm (Benjamini and Hochberg, 1995).

## Data Availability

The datasets presented in this study can be found in online repositories. The names of the repository/repositories and accession number(s) can be found below: https://www.ncbi.nlm.nih.gov/bioproject/ PRJNA950470.

## Supplementary material

SUPPLEMENTARY FIGURE S1 Lung microbial abundances. *Staphylococcus aureus* was the most abundant bacteria found in COVID-19-infected lung tissue, which occurred in both groups. From the eight samples of the control group, four showed increased relative abundance of *Streptococcus*, *Paeniclostridium*, *Escherichia*, and *Shigella.* From the six samples of the COVID-19 group, three showed increased relative abundance of specific genera, *Staphylococcus*, *Streptococcus*, and *Paeniclostridium*.

SUPPLEMENTARY FIGURE S2 Heart microbial abundances. The highest percentage of bacteria in heart tissue on the genera level was *Enterobacteriaceae* (*Escherichia* and *Shigella*), which occurred in both groups.

SUPPLEMENTARY FIGURE S3 Brain tax abundances. The highest percentage of bacteria in brain tissue on the genera level was *Enterobacteriaceae* (*Escherichia* and *Shigella*), which occurred in both groups.
