# Supplementary figures and images for: COVID-19 and brain-heart-lung microbial fingerprints in Italian cadavers
Source: Front Mol Biosci. 2023 Jun 14;10:1196328. doi: 10.3389/fmolb.2023.1196328 (PMC10300556; doi:10.3389/fmolb.2023.1196328)

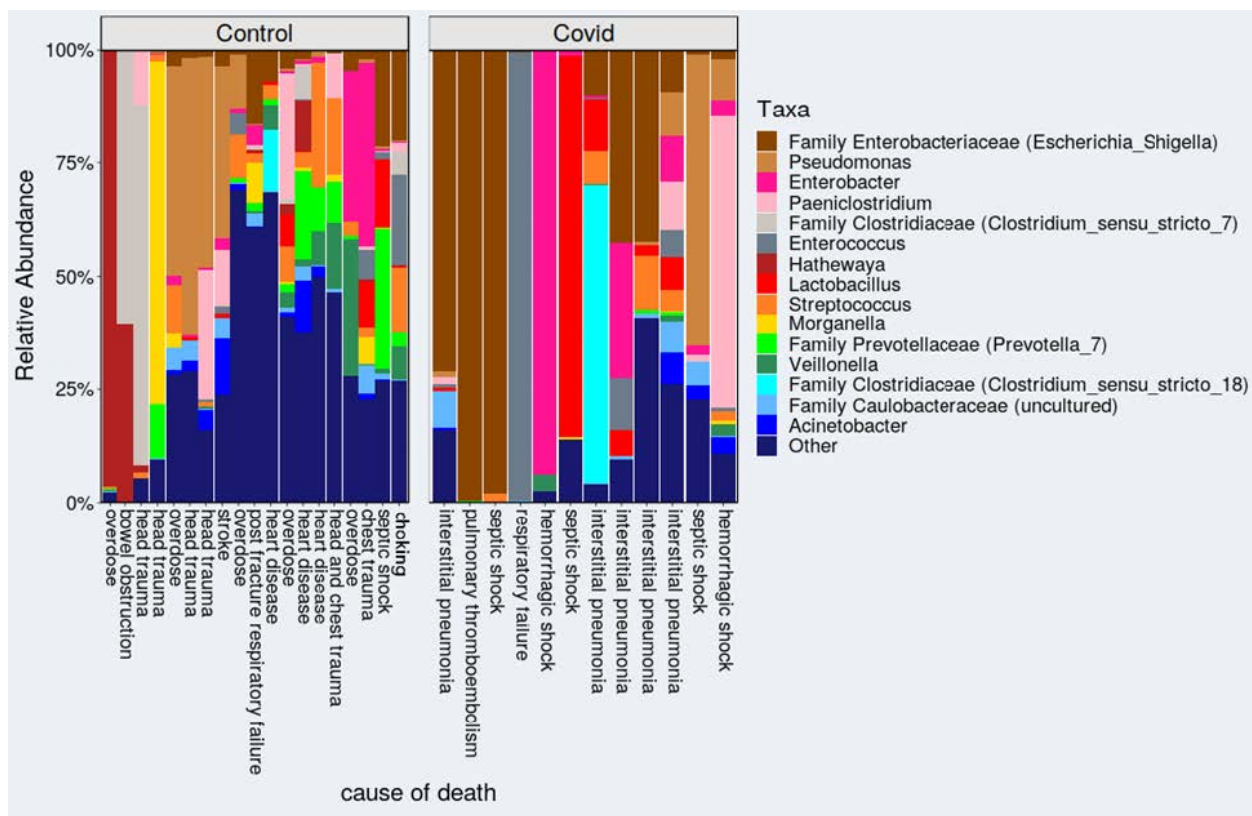

Supplement: Supplementary file 1 [file Image2.pdf]

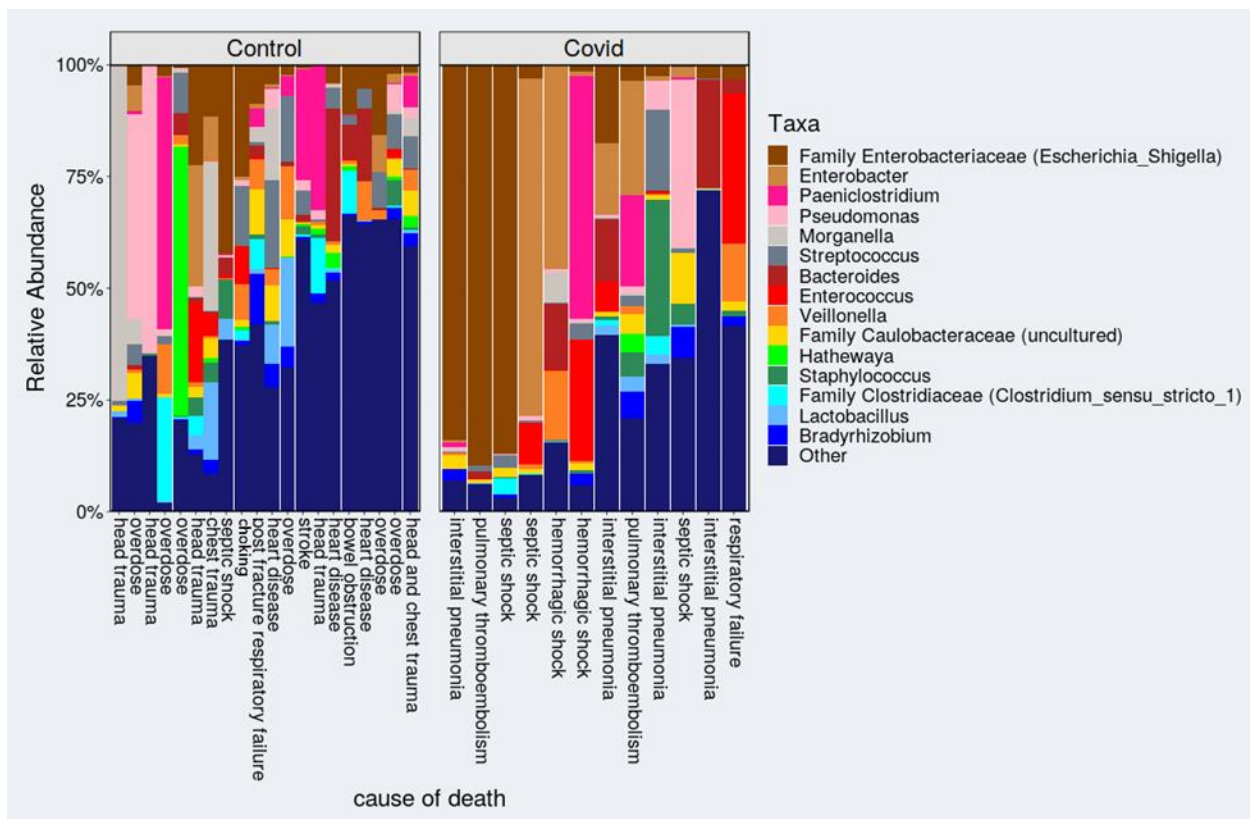

Supplement: Supplementary file 2 [file Image3.pdf]

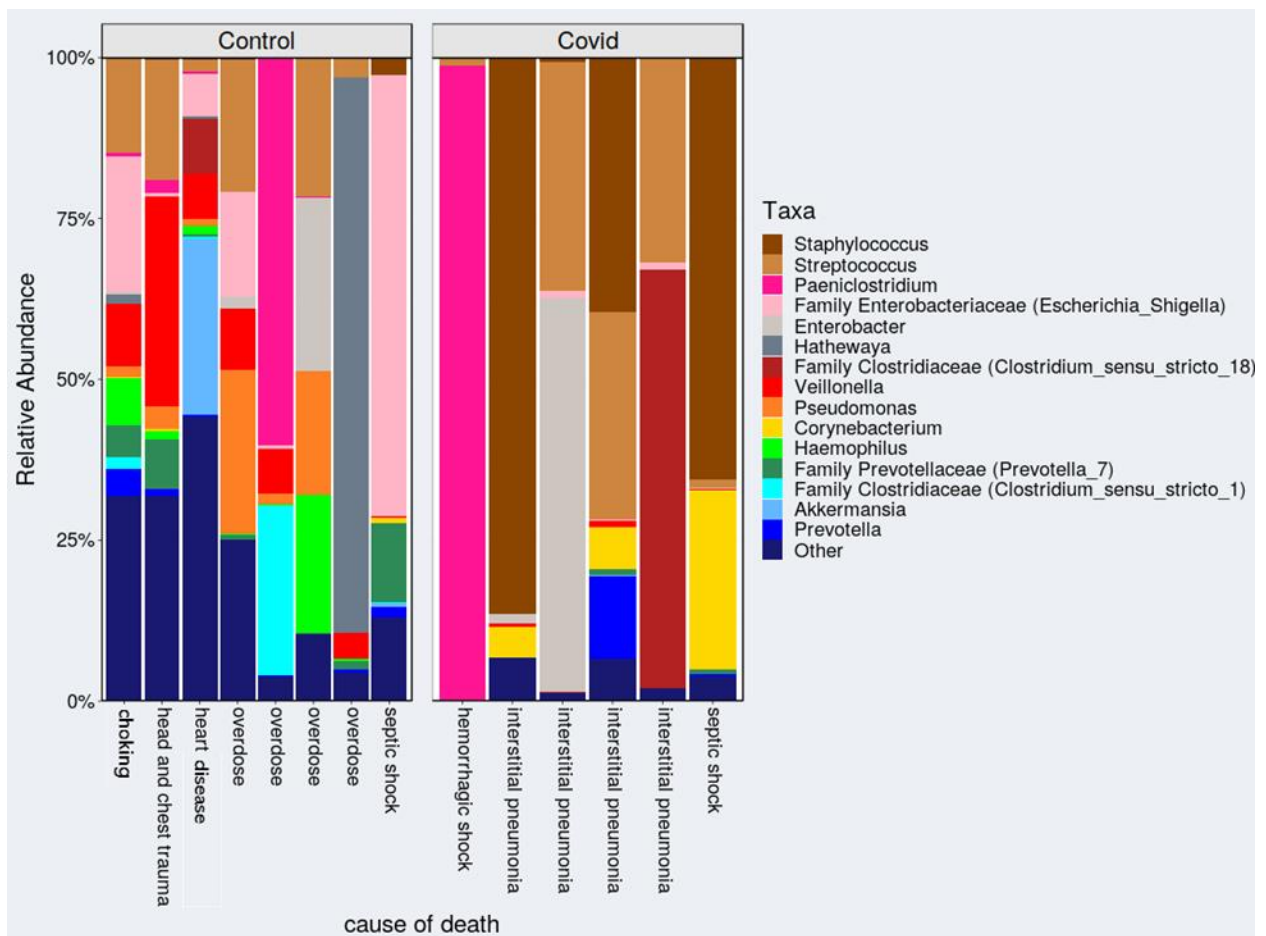

Supplement: Supplementary file 3 [file Image1.pdf]
